# Supplementary material for: Deletion of wfs1 Impairs Oligodendrocyte Precursor Cells Dorsal Distribution and Myelination Through the wfs1-hmgcs1 Axis in Zebrafish
Source: Biology (Basel). 2026 Aug 16;15(16):1402. doi: 10.3390/biology15161402 (PMC13510151; doi:10.3390/biology15161402)
Supplement: Supplementary file 1 [file biology-15-01402-s001.zip › File S1-Transcriptome sequencing data and differentially expressed genes (DEGs) in wfs1-- zebrafish at 5 dpf/2.KEGG/wfs1KOvsWT/GSEA/WT/BASAL_TRANSCRIPTION_FACTORS/BASAL_TRANSCRIPTION_FACTORS(DRE03022).html]

Details for gene set BASAL\_TRANSCRIPTION\_FACTORS(DRE03022)[GSEA]

|  || Dataset | deg.sample |
| Phenotype | sample.cls |
| Upregulated in class | WT |
| GeneSet | BASAL\_TRANSCRIPTION\_FACTORS(DRE03022) |
| Enrichment Score (ES) | -0.25383222 |
| Normalized Enrichment Score (NES) | -1.125662 |
| Nominal p-value | 0.20396039 |
| FDR q-value | 0.54908293 |
| FWER p-Value | 1.0 |
Table: GSEA Results Summary

  

Fig 1: Enrichment plot: BASAL\_TRANSCRIPTION\_FACTORS(DRE03022)      
 Profile of the Running ES Score & Positions of GeneSet Members on the Rank Ordered List

  

| PROBE | DESCRIPTION (from dataset) | GENE SYMBOL | GENE\_TITLE | RANK IN GENE LIST | RANK METRIC SCORE | RUNNING ES | CORE ENRICHMENT || 1 | 798997\_1 | gtf2a2 |  |  | 284 | 3.718 | 0.1207 | No |
| 2 | 798997 | gtf2a2 |  |  | 1691 | 1.546 | 0.1304 | No |
| 3 | 567899\_1 | gtf2e1 |  |  | 2736 | 1.101 | 0.1360 | No |
| 4 | 407713 | tbpl2 |  |  | 2909 | 1.057 | 0.1674 | No |
| 5 | 325832 | gtf2f1 |  |  | 3762 | 0.875 | 0.1712 | No |
| 6 | 450080 | mnat1 |  |  | 4196 | 0.794 | 0.1853 | No |
| 7 | 564106 | si:dkey-219c3.2 |  |  | 6156 | 0.572 | 0.1437 | No |
| 8 | 386637\_1 | taf12 |  |  | 6176 | 0.570 | 0.1630 | No |
| 9 | 324323 | ercc3 |  |  | 7211 | 0.473 | 0.1470 | No |
| 10 | 100150678 | taf4b |  |  | 8329 | 0.411 | 0.1262 | No |
| 11 | 81597\_1 | gtf2ird1 |  |  | 9021 | 0.366 | 0.1173 | No |
| 12 | 447818 | taf6 |  |  | 9061 | 0.363 | 0.1287 | No |
| 13 | 324486 | taf2 |  |  | 10180 | 0.292 | 0.1038 | No |
| 14 | 334854\_2 | gtf2e2 |  |  | 11208 | 0.233 | 0.0796 | No |
| 15 | 767629 | gtf2a1l |  |  | 13156 | 0.132 | 0.0231 | No |
| 16 | 557363 | taf6l |  |  | 13824 | 0.100 | 0.0057 | No |
| 17 | 368882 | tbp |  |  | 14727 | 0.059 | -0.0206 | No |
| 18 | 100149942 | taf4a |  |  | 17068 | -0.035 | -0.0928 | No |
| 19 | 556710 | taf10 |  |  | 17549 | -0.056 | -0.1060 | No |
| 20 | 324153\_1 | taf13 |  |  | 18432 | -0.095 | -0.1304 | No |
| 21 | 447857 | gtf2h1 |  |  | 18522 | -0.100 | -0.1297 | No |
| 22 | 751713\_1 | gtf2h5 |  |  | 18626 | -0.105 | -0.1293 | No |
| 23 | 334474 | taf3 |  |  | 18737 | -0.110 | -0.1289 | No |
| 24 | 324823 | gtf2b |  |  | 19693 | -0.152 | -0.1536 | No |
| 25 | 323974 | gtf2a1 |  |  | 19912 | -0.162 | -0.1548 | No |
| 26 | 334839 | gtf2h4 |  |  | 20549 | -0.191 | -0.1681 | No |
| 27 | 334055 | taf5l |  |  | 20622 | -0.195 | -0.1636 | No |
| 28 | 555452 | taf1 |  |  | 21124 | -0.222 | -0.1716 | No |
| 29 | 321485 | taf9 |  |  | 23081 | -0.333 | -0.2214 | No |
| 30 | 393900 | ercc2 |  |  | 23690 | -0.372 | -0.2275 | No |
| 31 | 436821 | taf11 |  |  | 23800 | -0.380 | -0.2177 | No |
| 32 | 100148942 | gtf2f2b |  |  | 24943 | -0.443 | -0.2381 | Yes |
| 33 | 323239 | gtf2h2 |  |  | 25307 | -0.467 | -0.2332 | Yes |
| 34 | 436837\_1 | gtf2h3 |  |  | 25966 | -0.526 | -0.2355 | Yes |
| 35 | 334854 | gtf2e2 |  |  | 26465 | -0.575 | -0.2311 | Yes |
| 36 | 393256 | taf8 |  |  | 26632 | -0.591 | -0.2157 | Yes |
| 37 | 541325 | ccnh |  |  | 26657 | -0.593 | -0.1958 | Yes |
| 38 | 641491 | taf5 |  |  | 27044 | -0.633 | -0.1858 | Yes |
| 39 | 334854\_1 | gtf2e2 |  |  | 27327 | -0.651 | -0.1720 | Yes |
| 40 | 100002261 | taf7 |  |  | 27766 | -0.711 | -0.1609 | Yes |
| 41 | 405897 | cdk7 |  |  | 27781 | -0.713 | -0.1365 | Yes |
| 42 | 403035 | tbpl1 |  |  | 28183 | -0.770 | -0.1223 | Yes |
| 43 | 415217 | gtf2f2a |  |  | 30006 | -1.248 | -0.1360 | Yes |
| 44 | 567899 | gtf2e1 |  |  | 31220 | -2.265 | -0.0951 | Yes |
| 45 | 798997\_2 | gtf2a2 |  |  | 31619 | -3.331 | 0.0085 | Yes |
Table: GSEA details [plain text format]

  

Fig 2: BASAL\_TRANSCRIPTION\_FACTORS(DRE03022)      
 Blue-Pink O' Gram in the Space of the Analyzed GeneSet

  

Fig 3: BASAL\_TRANSCRIPTION\_FACTORS(DRE03022): Random ES distribution      
 Gene set null distribution of ES for **BASAL\_TRANSCRIPTION\_FACTORS(DRE03022)**
